# Current Status and Prospects of Targeted Therapy for Osteosarcoma

**DOI:** 10.3390/cells11213507

**Published:** 2022-11-05

**Authors:** Zunguo Hu, Shuang Wen, Zijun Huo, Qing Wang, Jiantao Zhao, Zihao Wang, Yanchun Chen, Lingyun Zhang, Fenghua Zhou, Zhangyu Guo, Huancai Liu, Shuanhu Zhou

**Affiliations:** 1 Department of Joint Surgery, Affiliated Hospital of Weifang Medical University, School of Clinical Medicine, Weifang Medical University, Weifang 261061, China; 2 Department of Histology and Embryology, School of Basic Medical Sciences, Weifang Medical University, Weifang 261053, China; 3 Neurologic Disorders and Regenerative Repair Laboratory, Weifang Medical University, Weifang 261053, China; 4 Department of Orthopedic Surgery, Brigham and Women’s Hospital, Harvard Medical School, Boston, MA 02115, USA

**Keywords:** osteosarcoma, targeted therapy, chemotherapy, surgical treatment

## Abstract

Osteosarcoma (OS) is a highly malignant tumor occurring in bone tissue with a high propensity to metastasize, and its underlying mechanisms remain largely elusive. The OS prognosis is poor, and improving the survival of OS patients remains a challenge. Current treatment methods such as surgical approaches, chemotherapeutic drugs, and immunotherapeutic drugs remain ineffective. As research progresses, targeted therapy is gradually becoming irreplaceable. In this review, several treatment modalities for osteosarcoma, such as surgery, chemotherapy, and immunotherapy, are briefly described, followed by a discussion of targeted therapy, the important targets, and new technologies for osteosarcoma treatment.

## 1. Introduction

Osteosarcoma (OS) is the most common primary bone tumor, with a bimodal age distribution. The highest incidence is in children and adolescents, and the second highest is among older adults (>60 years) [1]. OS often occurs in the growth plate near the epiphysis of long bones; about two-thirds of tumors appear in the distal femur periapical to the knee. The next most common OS site is the proximal tibia, and the third most common site is the proximal humerus. Between 10% and 15% of patients with recently diagnosed OS have metastases [2,3]. Among patients with metastatic OS, lung metastasis is the most common (around 74%), followed by bone metastasis alone (about 9%), and about 8% of patients have both bone and lung metastasis [4]. Most standard treatments were established in the 1980s. These treatments, including surgery and chemotherapy, can achieve long-term survival in approximately 60% of patients with localized OS [5]. However, since the 1980s, there have been no new breakthroughs in OS treatment. As the biology of OS has come to be better understood, its heterogeneity and potential molecular aberrations have been revealed. However, in the face of inoperable surgery, we are still limited in what we can do for OS patients who are insensitive or intolerant to chemotherapy drugs. Resistance to chemotherapeutic drugs is also a problem that hinders breakthroughs in OS treatment [6]. The stagnation of research on the molecular mechanisms of OS also prevents us from making any new breakthroughs. With the recent advancements in molecular profiling technology for OS, we can look for targeted drugs to improve survival. In this review, we provide an overview of currently available surgery, chemotherapy, immunotherapy, and targeted therapies for the treatment of OS, as well as some new technologies for OS treatment, and we explore barriers to breakthroughs in OS treatment, focusing on popular OS-targeted pathways and their associated agents.

## 2. Surgical Treatment of OS

**Figure 1:**
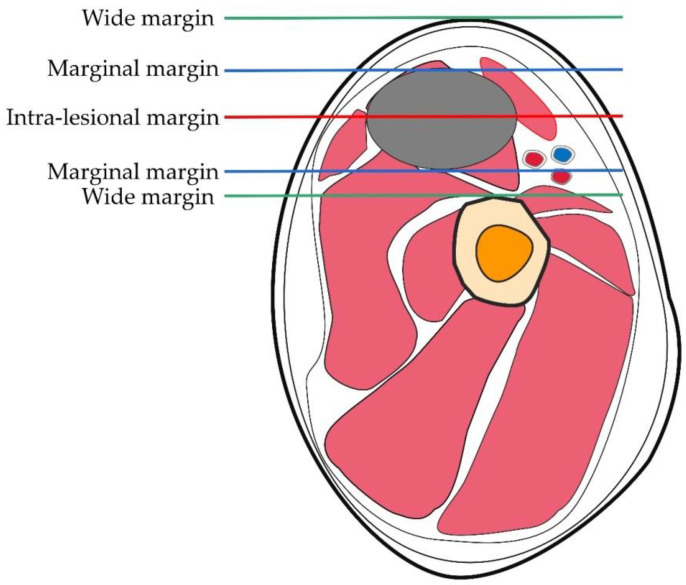
MSTS system for OS surgical resection margins. An intra-lesional margin is formed when any point enters the tumor during OS resection. The red line represents the intra-lesional margin. Margins are formed when peeling during surgery stretches to or across the reaction zone surrounding the tumor. The blue line represents the marginal margin. Wide margins are created when there is no access to the reaction zone and the entire dissection passes through healthy tissue. The green line represents the wide margin.

Surgical resection remains important in the survival of patients with OS. Although the goal must be the complete removal of the tumor, the extent of resection is also critical. The growth pattern of OS is radial, forming a spherical mass. When it penetrates the bone cortex, it creates a pseudo-envelope layer called the “reaction zone”, which is formed by the compression of the muscles surrounding the OS. The tumor nodules invading the reaction zone are called “satellites” and represent microextensions of the primary mass. When treated surgically, the entire tumor tissue, including the reaction zone (satellites), must be completely removed. A classic study by Enneking from 1980 includes both the definition and the characteristics of the surgical margin [7]: intra-lesional, marginal, wide, or radical. An intra-lesion margin is formed when any point enters the tumor during OS resection. Margins are formed when peeling during surgery stretches to or across the reaction zone surrounding the tumor. Larger margins are created when there is no access to the reaction zone and the entire dissection passes through healthy tissue. Radical margins are created when the entire bony or myofascial block or the block containing the tumor is removed [8]. The surgical margin is illustrated in Figure 1. Enneking articulated the concept of surgical margins through the relationship of the tumor to surrounding tissue, and his concept of surgical margins was the basis for the development of his surgical staging system, which was subsequently adopted by the American Society for Musculoskeletal Tumors (MSTS). This is a surgical planning method of preoperative dissection with the aim of removing the tumor to preserve limb function. Another classification system is the American Joint Committee on Cancer (AJCC) R system based on the findings of the surgical margins of the postoperative pathological specimens. A recent study compared surgical margin distance in primary high-grade sarcoma not receiving neoadjuvant therapy using the AJCC R classification and MSTS. The differences between the two are listed in Table 1. The MSTS system has higher reliability and negative predictive value [9]. A new surgical staging system for primary high-grade osteosarcoma was discovered, named the Birmingham classification. The use of chemotherapy response and surgical margins in millimeters was more predictive of the development of local recurrence risk factors than the MSTS classification system. The Birmingham classification may represent an improved ability to predict local recurrence risk factors and survival in osteosarcoma patients receiving neoadjuvant chemotherapy [10]. Due to insufficient experiments to prove that the Birmingham classification is superior to the MSTS classification, the MSTS classification is still the preferred classification method for the treatment of osteosarcoma by clinicians. Regardless of the type of OS a patient has, adequate surgical margins are critical to achieve complete tumor resection and optimize prognosis [11,12]. When patients are treated with preoperative chemotherapy to obtain adequate surgical margins, there is no significant difference in outcomes between limb preservation and amputation [13,14]. If patients with OS are treated only surgically, the results have not been found satisfactory, despite the severity of the ablative techniques employed including amputations and disarticulations [15,16]. Chemotherapy drugs, therefore, play an important role in the survival of patients with OS.

## 3. Chemotherapy for OS

Chemotherapy has not only broadened the treatment options for OS, but has actually become an irreplaceable treatment on its own. MAP (high-dose methotrexate, doxorubicin/Adriamycin, and cisplatin/platinum) is the most widely used chemotherapy regimen. These three chemotherapeutic agents are currently the most effective against OS [17,18]. Over the past 40 years, patient outcomes have been stabilized by this three-drug regimen. After complete surgical resection, the application of MAP yields a 5 year event-free survival (EFS) of approximately 60–70% in non-metastatic OS patients [19]. There are currently clinical trials of different combinations of five chemotherapeutic agents (methotrexate, doxorubicin, cisplatin, eflornithine, and etoposide) effective in the treatment of OS, some of which have used the pathological response at the surgery to select consolidation therapy; however, no further advancement has been achieved [20,21]. In patients with recurrent or metastatic OS, surgical removal of the tumor has a beneficial effect on survival [22,23]. The efficacy of chemotherapy regimens consisting of ifosfamide and etoposide or gemcitabine and doxorubicin in patients with unresectable recurrent OS has recently been demonstrated [24,25]. However, data from phase II trials of several treatments have shown that such patients have a poor prognosis, with an overall EFS of only 12% at 4 months [25]. In addition, treatment outcomes for patients with resectable recurrences also remain unsatisfactory, with a median EFS of 4 months and a 2 year EFS of 12% [26]. In several clinical trials, adjuvant chemotherapy (the addition of high-dose ifosfamide with or without MAP) is of limited utility in patients with OS, yielding a poor histologic response [27]. For this reason, we need more effective drugs and treatment protocols to improve survival.

## 4. Immunotherapy for OS

Immunotherapy was born when William Coley first discovered that the use of bacterial toxins could cause tumors to subside [28]. Within the human immune system, there exist highly complex responses and interactions among immune cells, cytokines, foreign threats, autoantigens, and antibodies to achieve functions such as immune defense, immune surveillance, and immune dynamic homeostasis [29,30]. The elimination of tumor cells through the autoimmune pathway is the key to immunotherapy. The immune response to tumors is dominated by cellular immunity, and the killing of tumor cells by natural killer cells (NK) and natural killer T cells (NKT) is an important pathway. Innate immune cells other than NK cells, including eosinophils, basophils, and phagocytes, are also involved in tumor suppression [31,32]. In an analysis of the tumor microenvironment (TME) of OS, it was shown that the immune cell infiltrate contains macrophages and T cells [33,34]. The presence of tumor-infiltrating lymphocytes (TILs) also provides a new direction for the immunotherapy of OS. In an experiment using in vitro cell lines and an in vivo mouse model, it was shown that anti-PD-1 antibody can inhibit the increase of OS tumor volume and prolong the overall survival time by inhibiting Treg [35]. Immunotherapy is also eager to make breakthroughs in other directions, such as adoptive cell therapy, oncolytic viruses, tumor vaccines, and dendritic cells, and checkpoint inhibitors [36]. Although an increasing number of patients with OS are benefiting, immunotherapy for patients with recurrent and metastatic OS remains limited. Immunotherapy acts on a large range of cells, and the resulting cytotoxicity is an important limiting factor, with unavoidable damage to OS patients. Therefore, we may achieve better efficacy through the combined application of targeted therapy and immunotherapy.

## 5. Targeted Therapy for OS

The 21st century has already seen comprehensive technological progress in clinical medicine, and precision medicine has gradually entered the mainstream. The customization of therapies using biomarkers that predict the response (or resistance) to specific molecularly targeted therapies is a core concept of precision medicine. Such targeted drugs have greatly improved the effectiveness of oncology drug therapy while reducing toxic side effects. The combination of surgical resection and chemotherapy has improved the postoperative survival rate to some extent, but mortality remains high, and the emergence of targeted therapy has brought new hope [37]. Nevertheless, precision medicine faces many challenges. For example, many recurrent molecular alterations are difficult to treat, and in the context of multiple secondary genomic alterations, the alterations that constitute tumor relevance are often unclear. Such factors have hindered the progress of OS treatment; therefore, collaboration among related organizations is needed to facilitate the progress of related research. Osteosarcoma-focused organizations have been established around the world, such as the US-based Children’s Oncology Group (COG), the UK-based Bone Cancer Research Trust, and the EU’s EuroBoNeT. These collaborative organizations will allow us to better investigate the mechanisms underlying OS and better treat patients. A COG data analysis revealed that the outcomes of patients with recurrent, unresectable OS have long remained unchanged, as reflected in phase II trials of a multitude of drugs [38]. This justifies the use of historical controls, which makes more clinical trials feasible and has important implications for the efficacy of new therapies for a rare disease like OS. These tissue libraries were also used in a large number of studies on the molecular analysis of OS. In recent years, several genomic studies have been performed using whole-genome sequencing or whole-exome sequencing, yielding a deeper understanding of the pathophysiology and genetics of the disease. Analysis of the OS genome has identified many genes associated with genetic heterogeneity, chromosomal abnormalities, and mutations [39,40]. The discovery of these genomes is of great significance for the targeted treatment of OS, as it provides more relevant targets and more targeted therapy options.

The molecular mechanisms of OS have remained a difficult research area for many years, although more pathway targets have been discovered along the way. Unlike other tumor types, OS lacks the high frequency of activating mutations in signaling genes. In OS, an equivalent gain of function follows from gene amplification and overexpression. Several candidate pathways containing targetable genes have been identified through the integration of gene copy number and expression data. These include the phosphatidylinositol 3-kinase (PI3K)–mammalian target of rapamycin (mTOR) pathway (PIK3CA, mTOR, and AKT1), the insulin-like growth factor (IGF) pathway (IGF1R), the vascular endothelial growth factor (VEGF) pathway (VEGFA and KDR), the platelet-derived growth factor (PDGF) pathway (PDGFR), the KIT and MYC pathways, and the cell-cycle pathway (CDK4, CCNE1, and CCND2), many of which are targetable by available agents [41,42,43]. The discovery of the newest therapeutic target, Wnt, also provides a powerful tool for the treatment of OS [44]. With the exception of the PI3K–mTOR pathway, which is most often activated by loss of phosphatase and tensin homolog deleted on chromosome ten (PTEN), recently nominated candidate drug targets predominantly fall in regions of frequent gene amplification. Given the rarity of OS and the large number of candidate genes that can be nominated by copy number and gene expression studies, discerning preclinical studies will be necessary to identify the best biomarkers for clinical validation. MYC oncogene amplification, which has been significantly associated with adverse outcomes, is being considered as a candidate biomarker for investigation in upcoming clinical trials [45]. The instability of the OS genome may entrain vulnerabilities that can be exploited. As compared with the genomes of cancers from other disciplines, OS genomes have a relatively high-level, homologous recombination-deficient signature (typically characteristic of BRCA1/2-deficient cancers), suggesting that poly ADP-ribose(adenosine diphosphate–ribose) polymerase (PARP) inhibitors, which are active in cancers associated with BRCA1/2 mutation, may have activity in OS [46,47]. Patients with Li–Fraumeni syndrome and hereditary retinoblastoma typically suffer from OS, allowing for the exploration of genetic susceptibility to OS and the tumor suppressor gene alterations of tumor protein p53 (TP53) and retinoblastoma 1 (RB1) that are prevalent in this malignancy [48,49]. Several rare genetic syndromes caused by defects in DNA helicases (RECQL4, WRN, and BLM) also increase the risk of OS [50,51]. In a recent study of the germline genetic structure of 1244 OS patients, known or potentially oncogenic variants or deleterious variants of proven human disease-causing genes were identified in approximately one-quarter of patients. Among the mutations identified, the most frequent was TP53 [52]. Table 2 lists the genes associated with somatic mutations in osteosarcoma-related pathways that have been implicated in the development of OS [53]. Targeted treatment of known OS genetic alterations will have an extremely important impact on the development of new OS treatments.

## 6. Different Targets for the Treatment of OS

### 6.1. Targeting DNA Damage Repair and Cell Cycle

**Figure 2:**
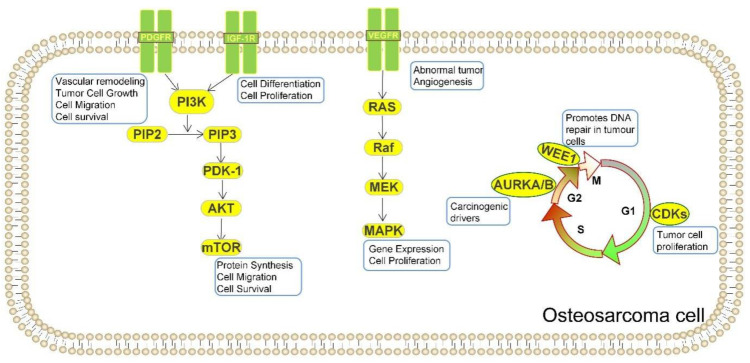
Important targets for osteosarcoma therapy and their main functions. Various receptors capable of acting as targets in OS cells serve different purposes. PDGFR promotes the survival and migration of OS cells, IGF-1 receptor promotes their differentiation; these two receptors can work together to produce mTOR in the PI3K pathway to enable OS cells to proliferate and metastasize. In addition to its vasculogenic role, VEGFR activates MEK, which has the effect of promoting OS gene expression. In addition to its angiogenic effects, VEGFR activates MEK, which also makes a useful contribution in promoting OS gene expression. Targets that also have important roles in the cell cycle, such as WEE1 acting at the G2/M test site, AURKA/B acting in mitosis, and CDKs produced in the G1 phase, show facilitative effects on OS.

Most cancer cells are harmful to the DNA damage response (DDR) and allow cancer cells to increase much more rapidly than most normal cells. The most common current treatment for cancer is radiation and chemotherapy that work by producing DNA damage, suggesting that most cancer cells are damaged by DDR. Thus DNA repair mechanisms as therapeutic targets can be used to fight cancer [59]. Several subpopulations of OS effect somatic changes through their influence on cell cycle and/or DNA damage repair pathways. For example, p35 deletion is a major factor in cancer development; hence, when TP53 is produced abnormally, it affects the associated G1 checkpoint of the cell cycle, thus increasing the dependence of tumor cells on the G2 cycle checkpoint to maintain DNA integrity in order to complete cell division [60]. Therefore, drugs that destabilize the G2 checkpoint, such as WEE1 inhibitors, may bolster DNA-damaging agent activity and initiate the death of TP53-mutated OS cells [61]. In preclinical trials in OS, the therapeutic activity of the WEE1 inhibitor AZD1775 alone was not significant, since it primarily acts as a sensitizer to the drug rather than a monotherapy on its own. Therefore, the combination with irinotecan, a topoisomerase 1 inhibitor, achieved good results not only in preclinical animal trials but also in phase I clinical trials [61,62]. CDK2 has been identified as an additional potential molecular target related to the cell cycle [63]. CDK4 and AURKA/B have also been found to have potential targets for OS treatment. It has been shown that CDK4 is overexpressed in OS patient-derived xenograft (PDX) models, human OS cell lines, and patient samples. In vitro inhibition of CDK4 by palbociclib inhibited growth of cell lines expressing high levels of CDK4 [64,65]. In humans, single-agent palbociclib caused some degree of tumor regression in a cisplatin-resistant PDX model, but the addition of sorafenib caused the tumor to subside further [66]. Dinaciclib has also been found to kill OS cells by inactivating CDK1 and CDK2. Dinaciclib in combination with a heat shock protein 90 inhibitor induces apoptosis of OS cells prepared from localized and metastatic tumors and other sarcoma cell types, but not normal osteoblasts or fibroblasts [67]. AURKA/B was also shown to be overexpressed in samples from patients with OS [68]. Inhibitors of AURKA/B, such as the AURKA inhibitor Alisertib and the AURKB inhibitors AZD1152 and HoI-07, have been studied for their therapeutic effects on OS [69,70]. In an experiment with two human OS cell lines, U-2OS and MG-63, the AURKA inhibitor Alisertib could induce autophagy in OS cells by activating the mitochondria-dependent apoptotic pathway [69]. According to the study of human osteosarcoma cell lines, 143B, KHOS, U-2OS, MG-63, and SaoS-2, and the osteoblast cell line, hFOB 1.19, the expression of Aurora B kinase in OS is higher than that in normal tissues, and its specific inhibitor HOI-07 significantly inhibited OS cell proliferation and induced apoptosis; no significant toxicity with HOI-07 was observed in the PDX mouse model [70]. Figure 2 depicts the period of the cell cycle in which it acts and the main role it plays. The DDR pathway is an important target for OS treatment due to the many somatic cell alterations found in patient samples. As previously mentioned, most mutations in somatic cells are in the form of chromosome breaks and translocations rather than point mutations, suggesting a defect in the DDR machinery [71]. DDR plays an important role in the mechanism of OS formation, suggesting that, by better understanding the process of DNA damage response, it may provide us with additional therapeutic targets in terms of treatment and prognosis, which could improve our current therapeutic approach.

### 6.2. Targeting Vascular Endothelial Growth Factor

Vascular endothelial growth factor (VEGF) is essential in angiogenesis, with not only a major regulatory role in angiogenesis, tumor growth, and development, but also a significant regulatory role with different cell types, particularly endothelial cells. At the same time, it also plays a major role in the vascular homeostasis of various tissues and the molecular pathogenesis of tumor growth and metastasis [72]. The expression of VEGFA in OS is associated with a higher risk of pulmonary metastasis and poorer survival. Meanwhile, an experiment with tissue sections from OS patients showed that VEGFA gene amplification predicts poorer tumor-free survival [73,74]. These findings have stimulated research on targeted drugs acting on the vascular endothelial growth factor pathway. Thus, by inhibiting the signaling pathway of VEGF, the growth of OS cells can be inhibited and apoptosis of OS cells can be promoted [72]. It is hoped that exploiting this pathway will improve both prognosis and survival of patients with OS. Drugs shown to improve outcomes through this route include regorafenib, cabozantinib, lenvatinib, and sorafenib. Cabozantinib has good antitumor activity and was well tolerated in a phase II trial in patients with osteosarcoma, and it may be investigated as a new treatment option for OS [75]. Lenvatinib is also being investigated as a VEGFR-targeted drug in OS, and lenvatinib monotherapy has also shown antitumor activity in a phase II clinical trial with 31 OS patients [76,77]. Regorafenib has not only achieved good results in terms of single-agent activity, but also achieved good results in phase II clinical trials for metastatic OS [78,79]. Regorafenib is also recommended in the NCCN guidelines as a first-line treatment for patients with relapsed or refractory OS. The VEGF pathway has an ideal progress in the treatment of OS, and the drugs produced through the exploration of this pathway have significant activity in OS. The exploration of this pathway can be used as the focus of the treatment of OS patients, and in the future, with the continuous progress of research, there can be expected results to be discovered.

### 6.3. Targeting Platelet-Derived Growth Factor

The signaling system of platelet-derived growth factor (PDGF) is composed of four ligands PDGF-A, PDGF-B, PDGF-C, and PDGF-D, and two receptors, PDGF-α and PDGF-β [80,81,82]. Four ligands composed of polypeptide chains in the PDGF signaling system have been found to constitute the five functional growth factors PDGF-AA, PDGF-BB, PDGF-AB, PDGF-CC, and PDGF-DD [83]. PDGF acts as a mitogenic factor in OS cell lines, and PDGF-AA and platelet-derived growth factor receptor (PDGFR)-α are co-expressed in OS and associated with poor prognosis [50]. Studies suggest that PDGF is a therapeutic target for OS. Imatinib is a tyrosine kinase inhibitor that targets binding to c-kit and PDGF receptors (PDGFR). Imatinib inhibits PDGF-mediated growth and apoptosis of OS cell lines in vitro. Despite promising results in in vitro testing, the single use of imatinib in phase II clinical trial of the COG study did not produce a corresponding effect against OS [84,85]. Similarly, recent studies have shown that targeting PDGFR is not sufficient to control the growth of OS, especially in the presence of other growth-promoting factors. However, in a mouse model established by human OS cell lines SAOS2, SJSA1, and U2OS, it was shown that, under the induction of Adriamycin (ADR), not only did imatinib have a synergistic antiproliferative effect in vitro, it also had a therapeutic effect in the body [86]. Most drugs targeted to the PDGF pathway relevant to the treatment of OS are multitarget receptor tyrosine kinase inhibitors that block not only PDGFR but also VEGFR, such as apatinib, anlotinib, sunitinib, and sorafenib [87,88]. Studies of sunitinib in a 143B cell-derived osteosarcoma model in server combined immune-deficiency (SCID) mice showed that it not only slowed tumor growth, but also inhibited lung metastasis [89]. The PDGF pathway plays an important role in the treatment of other tumors. Although the use of drugs targeting this pathway in OS has not achieved satisfactory results, it is closely related to VEGF and still has the potential to be continuously explored.

### 6.4. Targeting Insulin-like Growth Factor

The insulin-like growth factor (IGF) system is not only associated with the proliferation and differentiation of osteoblasts, but also has a vital role in bone formation and dynamic homeostasis [90,91]. Differential expression of IGF-I and IGF-II and more consistent expression of IGF-1R are observed in OS tissue samples [92,93]. Overexpression of IGF- I and IGF-1R is associated with tumorigenesis and is considered a risk factor for the development of many human cancers [94]. IGF- I/IGF-1R signaling has been extensively studied in tumors [95,96]. Meanwhile, in investigations of IGF-1R expression, increased expression of IGF-1R was closely associated with malignant metastasis and prognosis of OS, and high expression of IGF-1R in OS patients was associated with poorer prognosis [97]. In addition, inhibition of growth hormone release in mice by growth shift analogs or growth hormone-releasing hormone antagonists reduces serum IGF-1 levels and inhibits tumor growth, possibly because these compounds reduce IGF-I stimulation and directly affect cell growth [98,99]. However, a phase I clinical trial of long-acting somatostatin analogs in OS patients showed that, although long-acting somatostatin analogs can reduce serum IGF-I levels, no clinical response was observed [100]. The IGF-1R inhibitor R1507 had low response rates and limited activity in a phase II clinical trial in 38 patients with OS [101,102]. Although the IGF pathway is considered to be one of the drivers of OS, there is no obvious effect of blocking this pathway as we envision. This suggests that other substances play a role in IGF in the process of OS development. Research on the IGF pathway has not achieved matching clinical effects, and more in-depth studies of this pathway are needed to obtain reliable and beneficial treatment options for patients.

### 6.5. Targeting PI3K/mTOR

Pathway analysis of mutated genes, clinical interpretation of individual genomes, and genomic screens have demonstrated the dependence of OS on PI3K/mTOR pathway activation [42]. The mammalian target of rapamycin (mTOR) pathway is important not only for mesenchymal stem cells but also in bone biology. This pathway is considered a key target for the treatment of OS [103], as its activation promotes the proliferation and metastasis of OS cells and inhibits intracellular apoptosis and autophagic processes. The mTOR inhibitor can inhibit the growth of OS cells in vitro and in vivo, and targeting this pathway can inhibit the growth of tumor cells [104,105]. Dual PI3K/mTOR inhibitors were also found to be effective in the treatment of OS in both in vitro osteosarcoma cell experiments and in vivo mouse experiments, and this antitumor effect could be enhanced by MEK inhibitors [106,107,108]. The targets of action associated with this pathway and the main functions of action of mTOR are presented in Figure 2. Therapy targeted to this pathway not only has good therapeutic effects when used alone, but also enhances the antitumor effect when used in combination with other drugs for the treatment of OS. In a trial of the human OS cell lines HOS, U2OS, and MG-63, Buparlisib showed not only significant efficacy as a single agent, but also good antitumor effects in combination with doxorubicin and vincristine [109]. In an experiment with human osteosarcoma MG-63 cells, rapamycin targets this pathway by activating autophagy, inhibiting tumor cell proliferation and promoting OS tumor cell apoptosis [110]. It was also shown that rapamycin and everolimus can act synergistically to achieve synergistic antitumor effects in PDOX mouse model [111]. Recent studies have identified PTEN as a tumor suppressor gene that can negatively regulate the PI3K–mTOR pathway and perhaps become a new target for the treatment of OS patients [112]. ZIP10 has also been shown to activate this pathway by promoting CREB-mediated ITGA10, which has a significant function in the chemoresistance of OS cells [113]. Therefore, we are confident that research on this pathway is of great importance and that more important therapeutic targets can be identified as the mechanisms are further investigated.

### 6.6. Targeting MYC

Avian myelocytomatosis viral oncogene homolog (MYC) is one of the most common oncogenes known to be activated in human cancers, and its amplification often suggests a poor prognosis [114,115]. MYC plays a major role in cancer by promoting growth, cell-cycle progression, metabolism, and survival [116]. Therefore, research on MYC expression as a therapeutic target for cancer continues and has already shown promising results [117,118]. The expression of MYC and its prognostic significance also play an integral role in the therapeutic goals of OS [119]. There is evidence that high MYC expression has an important impact on the survival of OS patients by promoting metastasis and reducing survival [45]. There are three important members of the MYC gene family, C-Myc, N-Myc, and L-Myc, of which overexpression of C-Myc has been shown to be strongly associated with OS prognosis [120]. Proliferation and metastasis of OS cells can be inhibited by therapies such as targeting MYC super-enhancer inhibitors [120]. For example, THZ1 and JQ1 inhibited MYC-induced transcriptional amplification in OS by inhibiting super-enhancers of MYC target genes in several cell line experiments and xenograft tumor models in osteosarcoma [120,121]. Studies show that targeting this pathway can be effective in improving patient prognosis and can have a strong impact on patient survival. While precision medicine holds promise for OS treatment, many challenges remain. For example, most recurrent molecular alterations are difficult to treat, and in the context of multiple secondary genomic alterations, those that constitute tumor associations are often difficult to identify. Furthermore, clinical trials using pathway alterations as biomarkers of choice for patients with rare diseases such as OS are challenging. Since the advent of karyotyping techniques, OS has been thought to have a complex genomic structure [18]. Because all these factors have slowed the progress of OS treatment, further progress in OS research requires collaborative efforts among multiple organizations. The drugs targeting vital OS targets in the above pathways are listed in Table 3.

### 6.7. Targeting OS Surfaceome

Many overexpressed cell surface molecules are present on the surface of OS cells. Thus, it is possible to target these surface molecules with relevant studies for targeted therapy. Surface molecules aggregated on the surface of OS that are currently under investigation are glycoprotein non-metastatic melanoma protein B (GPNMB), leucine-rich repeat-containing protein 15 (LRRC15), B7-H3, human epidermal growth factor receptor-2 (HER2) and CD2. GPNMB, B7-H3, and LRRC15 were expressed in more than 90% OS specimens by immunohistochemistry [122,123,124]. GPNMB was shown to be expressed in OS by methods such as immunohistochemistry and ELISA, and the targeted drugs associated with it are not very effective in the corresponding clinical trials [125]. Glembatumumab vedotin, a targeted antibody–drug coupling to GPNMB, did not achieve meaningful efficacy in a clinical trial of recurrent or refractory OS in children. Further development of new drugs is still to be expected in this regard. In a pediatric preclinical trial, ABBV-085, an antibody-drug conjugate targeting LRRC15, showed significant antitumor activity against a PDX model with high LRRC15 expression [126]. The discovery of B7-H3 CAR T cells may lead to a new therapeutic option, and more exploration is needed to enable their use in treating patients. Recent studies on GD2 and HER2 have demonstrated that targeted therapy with either GD2-BsAb or HER2-BsAb can exert effective antitumor activity in OS patients. Moreover, the efficacy of GD2-BsAb or HER2-BsAb can be improved when combined with immune checkpoint inhibitors such as PD-L1 [127]. Although it was found to have good antitumor effects, continuous research is needed to develop drugs that can be used in the clinic. These proteins, which are enriched on the surface of OS cells, can provide more clinically applicable targets for treatment. At the same time, understanding of these surface proteins is still far from adequate. Further exploration will provide more directions for treating OS and bring targeted drugs with better effects.

## 7. New Treatment for OS

The treatment of OS is not limited to the above treatment methods, such as surgery, chemotherapy, immunotherapy, and targeted therapy, and there are still new treatments being developed. Photodynamic therapy is a new type of cancer therapy and has been shown to be effective in superficial tumors such as breast cancer, melanoma, and soft tissue sarcoma [128,129]. Photosensitizers, light sources, and oxygen are three key aspects of photodynamic therapy capable of producing antitumor effects [128]. Photodynamic therapy plays a role in the cytotoxic mechanisms of OS, such as in autophagy, apoptosis, necrosis, cell-cycle arrest, tumor vascular damage, and immunogenic cell death [130]. According to the study of OS photodynamic therapy using in vitro experiments and animal model experiments, photodynamic therapy can not only eradicate OS cells in cell culture, but there is also evidence that it has a certain curative effect in OS animal model experiments [131]. Moreover, nanotechnology-based advances have contributed to the development of OS, and they have provided new ways to overcome traditional therapies. The development of nanotechnology has allowed more nanomaterials to be used in the targeted therapy of OS, such as the application of graphene oxide nanomaterials [132]. Nanomaterials for the treatment of OS are also in the process of continuous research and development. Currently, the popular nanomedicine systems are polymeric nanocarriers, liposomes, metallic nanoparticles, redox-responsive nanoparticles, hybrid nanoparticles, mesoporous silica nanocarriers, and calcium phosphates nanocarriers [133]. As excellent carriers, nanoparticles can not only transport various small molecules or drugs to target sites, but also can be combined with photosensitizers to prepare nano-drug delivery systems for photodynamic therapy [131,134,135]. Mesenchymal stromal cells can internalize and deliver nanoparticles loaded with therapeutic drugs [136,137]. Lenna. et al. showed that mesenchymal stromal cells can deliver photosensitizer-loaded nanoparticles in vitro and in a murine in vivo ectopic OS model and inhibit the growth of OS [138]. These new technologies open new avenues for the direction of OS treatment.

## 8. Obstacles to OS Progress and Treatment

OS is a complex and heterogeneous tumor characterized by a high degree of genomic instability, aneuploidy, and genomic rearrangement, with the presence of partial chromosomal gains (1p, 1q, 6p, 8q, and 17p) or partial chromosomal deletions (3q, 6q, 9, 10, 13, 17p, and 18q) [139]. OS can also arise from inherited genetic disorders such as Li–Fraumeni syndrome (p53 mutation) or mutations in the gene encoding Rb, and Rothmund–Thomson (RECQL4 mutation), Bloom (BLM), and Werner (WRN) syndromes [53,140]. The complexity and diversity of OS itself not only bring difficulties to its diagnosis but also bring insurmountable obstacles to its treatment. At the same time, it also brings drug resistance during OS treatment and relapse after treatment [141]. Targeting specific driver genes is also problematic due to the high heterogeneity and low incidence of OS. Moreover, there are many reasons why the results of theoretical research cannot be immediately applied to clinical research. For example, CDK4/6 inhibitors play an important role in the treatment of breast cancer and liposarcoma [142,143]. Although CDK4/6 has been detected in osteosarcoma [42], because its isolated expansion is rare in high-grade OS, the design of clinical trials targeting this target is difficult [144]. The use of active targeting strategies, such as ligand-mediated tumor targeting, currently under investigation, could improve the ability of drugs to target cancer [145]. Being able to discover qualified ligands is critical in this process. Although significant progress in the creation of new multifunctional ligand chemistry platforms may hold great promise for the future treatment of OS, most of them are still at the stage of cell and animal experiments, and translating them into clinical trials can be applied. There is still a long transition period before the human body. Furthermore, how to directionally move targeted drugs to the binding site, how to remove residual cancer tissue after surgery, and how to deal with the existence of patients with poor chemotherapy effect or who cannot tolerate chemotherapy are issues that can create resistance to the progress of OS treatment. Not only are there obstacles to breakthroughs in traditional treatment methods, but there are also limitations to the development of some new technologies. The effectiveness of photodynamic therapy in patients with OS lung metastases, the penetration of some radiation wavelengths that can effectively activate the photosensitizer in the face of deep tissues where OS is located, and the more accurate targeting of the photosensitizer to OS tissues are current difficulties being faced. At present, more research results are needed to promote progress and development as to whether nanomaterials can be delivered to OS tissues after entering the human body, as well as the subsequent toxicity evaluation. Mesenchymal stromal cells, as a new tool in the treatment of OS, still need a long study before they can be truly applied to clinical patients. All are obstacles on the way to new breakthroughs in OS treatment.

## 9. Conclusions and Prospects

Improving the prognosis of patients with OS, including prolonging survival, has long been a major challenge. However, over the past decade, our understanding of OS has gradually increased. Through continuous research into molecular analysis techniques, OS has also been studied more thoroughly, and more progress has been made. Tissue banks and collaborations established by various institutions for this rare disease have also played an important role in such progress. The development of preclinical models and the establishment of the PDX model for OS have increased the likelihood of clinical trials for OS, which are crucial to facilitating the clinical evaluation of new therapies. Treatment for OS is no longer confined to surgery and chemotherapy but now includes approaches such as immunotherapy and targeted therapy. Among the important breakthroughs in targeted therapies, the discovery of pathways associated with OS development, in particular, has provided new therapeutic targets. However, mechanisms underlying various targets remain unclear, which continues to hinder research, as do the many unexplored side-effects of treatment. Thus, further research and exploration of the disease are required to improve patient outcomes and prognosis. The close integration and transformation of basic research and clinical research are also required to enable breakthroughs in treating OS. As new biological research and discoveries and new technologies emerge, there is the promise of a better understanding of OS and much greater progress in its treatment.

## Figures and Tables

**Table 1:** Comparison of two classifications of surgical margins for OS [9].

Classification	Sensitivity	Negative Predictive Value	Preoperative Planning Instructions
AJCCR	Generally	Usually	None
MSTS	Very	Better	Include

**Table 2:** Somatic mutations in cancer genes in osteosarcoma-associated signaling pathways.

Signaling Pathway	Genes	References
PI3K-mTOR	EGFR, GNAQ, ALK, PDGFRA, PDGFRB, CBL, PIK3CA, PIK3R1, PDPK1, AKT1, AKT2, E1F4B, PTEN, TSC2	[42,53]
DNA damage control	WRN, ATM, CDKN2A, TP53, BRCA1, BRCA2, MLH1, BAP1	[46,53,54]
RAS	EGFR, GNAQ, ALK, PDGFRA, PDGFRB, CBL, NF1	[53]
Cell cycle/apoptosis	CDK4, MDM2, MYC, CARD11, CTNND1, BLM, CCNE1, COPS3, PRKCA, TWIST1, TP53, CDKN2A	[55,56,57]
TGF	GNAS, EP300	[53,58]

**Table 3:** Drugs for each targeted pathway associated with osteosarcoma and their therapeutic targets.

Action Pathway	Drugs	Target of Action	References
DNA damage repair and cell cycle	AZD1775 Palbociclib Dinaciclib Alisertib AZD1152 HOI-07	WEE1 inhibitor CDK4/6 inhibitors multi-CDK inhibitors AURKA inhibitor AURKB inhibitor	[61] [65] [67] [69] [70] [70]
VEGF and PDGF	Sorafenib Cabozantinib Lenvatinib Regorafenib Anlotinib Sunitinib	VEGFR inhibitors VEGFR and PDGFR inhibitors	[66] [75] [76,77] [78] [88] [89]
IGF	R1507	IGF-1R inhibitor	[101]
PI3K/mTOR	Buparlisib Rapamycin Everolimus	PI3K inhibitor mTOR inhibitors	[109] [110] [109,111]
MYC	THZ1 JQ1	MYC super-enhancer inhibitors	[120] [121]

## Data Availability

Not applicable.

## Abbreviations

OS	Osteosarcoma
MSTS	Musculoskeletal tumors
AJCC	American Joint Committee on Cancer
EFS	Event-free survival
NK	Natural killer cell
NKT	Natural killer T cell
COG	Children’s Oncology Group
PI3K	Phosphoinositide-3 kinase
PIK3CA	Phosphatidylinositol 3-kinase
mTOR	Mechanistic target of rapamycin
IGF	Insulin-like growth factor
IGF-1R	Insulin-like growth factor 1 receptor
VEGF	Vascular endothelial growth factor
VEGFA	Vascular endothelial growth factor A
KDR	Kinase insert domain receptor
PDGF	Platelet-derived growth factor
PDGFR	Platelet-derived growth factor receptor
DDR	DNA damage response
KIT	KIT proto-oncogene receptor tyrosine kinase
MYC	Avian myelocytomatosis viral oncogene homolog
CDK	Cyclin-dependent kinase
CCNE1	Cyclin E1
CCND2	Recombinant human cyclin-D2
PTEN	Phosphatase and tensin homolog deleted on chromosome ten
ADP	Adenosine diphosphate
PARP	Poly ADP-ribose polymerase
TP53	Tumor protein p53
RB1	Retinoblastoma1
PDX	Patient-derived xenograft
PDOX	Patient-derived orthotopic xenograft
ADR	Adriamycin
SCID	Server combined immune-deficiency
MEK	Mitogen-activated protein kinase kinase
MAPK	Mitogen-activated protein kinase
GPNMB	Glycoprotein non-metastatic melanoma protein B
CAR-T	Chimeric antigen receptor T
HER2	Human epidermal growth factor receptor-2
BsAb	Bispecific antibody

## References

[1] Gill J., Ahluwalia M.K., Geller D., Gorlick R. (2012). New Targets and Approaches in Osteosarcoma. Pharmacol. Ther..

[2] Meltzer P.S., Helman L.J. (2021). New Horizons in the Treatment of Osteosarcoma. N. Engl. J. Med..

[3] Kager L., Zoubek A., Pötschger U., Kastner U., Flege S., Kempf-Bielack B., Branscheid D., Kotz R., Salzer-Kuntschik M., Winkelmann W. (2003). Primary Metastatic Osteosarcoma: Presentation and Outcome of Patients Treated on Neoadjuvant Cooperative Osteosarcoma Study Group Protocols. J. Clin. Oncol..

[4] Bielack S.S., Kempf-Bielack B., Delling G., Exner G.U., Flege S., Helmke K., Winkler K. (2002). Prognostic Factors in High-Grade Osteosarcoma of the Extremities or Trunk: An Analysis of 1,702 Patients Treated on Neoadjuvant Cooperative Osteosarcoma Study Group Protocols. J. Clin. Oncol..

[5] Isakoff M.S., Bielack S.S., Meltzer P., Gorlick R. (2015). Osteosarcoma: Current Treatment and a Collaborative Pathway to Success. J. Clin. Oncol..

[6] Hattinger C., Patrizio M., Fantoni L., Casotti C., Riganti C., Serra M. (2021). Drug Resistance in Osteosarcoma: Emerging Biomarkers, Therapeutic Targets and Treatment Strategies. Cancers.

[7] Enneking W.F., Spanier S.S., Goodman M.A. (1980). A System for the Surgical Staging of Musculoskeletal Sarcoma. Clin. Orthop. Relat. Res..

[8] Enneking W.F., Maale G.E. (1988). The Effect of Inadvertent Tumor Contamination of Wounds during the Surgical Resection of Musculoskeletal Neoplasms. Cancer.

[9] Cates M.M., Cates J.M.M. (2019). Surgical Resection Margin Classifications for High-Grade Pleomorphic Soft Tissue Sarcomas of the Extremity or Trunk: Definitions of Adequate Resection Margins and Recommendations for Sampling Margins from Primary Resection Specimens. Mod. Pathol..

[10] Jeys L.M., Thorne C.J., Parry M., Gaston C.L.L., Sumathi V.P., Grimer R.J. (2017). A Novel System for the Surgical Staging of Primary High-grade Osteosarcoma: The Birmingham Classification. Clin. Orthop. Relat. Res..

[11] Iwata S., Ishii T., Kawai A., Hiruma T., Yonemoto T., Kamoda H., Asano N., Takeyama M. (2013). Prognostic Factors in Elderly Osteosarcoma Patients: A Multi-Institutional Retrospective Study of 86 Cases. Ann. Surg. Oncol..

[12] Bertrand T.E., Cruz A., Binitie O., Cheong D., Letson D.G. (2015). Do Surgical Margins Affect Local Recurrence and Survival in Extremity, Nonmetastatic, High-grade Osteosarcoma?. Clin. Orthop. Relat. Res..

[13] Jauregui J.J., Nadarajah V., Munn J., Pivec R., Kapadia B.H., Lerman D.M., Maheshwari A.V. (2018). Limb Salvage Versus Amputation in Conventional Appendicular Osteosarcoma: A Systematic Review. Indian J. Surg. Oncol..

[14] Odri G.A., Tchicaya-Bouanga J., Yoon D.J.Y., Modrowski D. (2022). Metastatic Progression of Osteosarcomas: A Review of Current Knowledge of Environmental versus Oncogenic Drivers. Cancers.

[15] Zhou Y., Huang Z., Wu S., Zang X., Liu M., Shi J. (2014). Mir-33A Is Up-Regulated in Chemoresistant Osteosarcoma and Promotes Osteosarcoma Cell Resistance to Cisplatin by Down-Regulating TWIST. J. Exp. Clin. Cancer Res..

[16] Gianferante D.M., Mirabello L., Savage S.A. (2017). Germline and Somatic Genetics of Osteosarcoma—Connecting Aetiology, Biology and Therapy. Nat. Rev. Endocrinol..

[17] Meyers P.A. (2015). Systemic Therapy for Osteosarcoma and Ewing Sarcoma. Am. Soc. Clin. Oncol. Educ. Book.

[18] Anninga J.K., Gelderblom H., Fiocco M., Kroep J.R., Taminiau A.H., Hogendoom P.C., Egeler R.M. (2011). Chemotherapeutic Adjuvant Treatment for Osteosarcoma: Where Do We Stand?. Eur. J. Cancer.

[19] Meyers P.A., Schwartz C.L., Krailo M., Kleinerman E.S., Betcher D., Bernstein M.L., Conrad E., Ferguson W., Gebhardt M., Goorin A.M. (2005). Osteosarcoma: A Randomized, Prospective Trial of the Addition of Ifosfamide and/or Muramyl Tripeptide to Cisplatin, Doxorubicin, and High-Dose Methotrexate. J. Clin. Oncol..

[20] Ferrari S., Ruggieri P., Cefalo G., Tamburini A., Capanna R., Fagioli F., Comandone A., Bertulli R., Bisogno G., Palmerini E. (2012). Neoadjuvant Chemotherapy with Methotrexate, Cisplatin, and Doxorubicin with or without Ifosfamide in Nonmetastatic Osteosarcoma of the Extremity: An Italian Sarcoma Group Trial ISG/OS-1. J. Clin. Oncol..

[21] Gaspar N., Occean B.V., Pacquement H., Bompas E., Bouvier C., Brisse H.J., Brugieres L. (2018). Results of Methotrexate-Etoposide-Ifosfamide Based Regimen (M-EI) in Osteosarcoma Patients Included in the French OS2006/Sarcome-09 Study. Eur. J. Cancer.

[22] Briccoli A., Rocca M., Salone M., Guzzardella G.A., Balladelli A., Bacci G. (2010). High Grade Osteosarcoma of the Extremities Metastatic to the Lung: Long-Term Results in 323 Patients Treated Combining Surgery and Chemotherapy, 1985–2005. Surg. Oncol..

[23] Ballatori S.E., Hinds P.W. (2016). Osteosarcoma: Prognosis Plateau Warrants Retinoblastoma Pathway Targeted Therapy. Signal Transduct. Target. Ther..

[24] Palmerini E., Jones R.L., Marchesi E., Paioli A., Cesari M., Longhi A., Meazza C., Coccoli L., Fagioli F., Asaftei S. (2016). Gemcitabine and Docetaxel in Relapsed and Unresectable High-Grade Osteosarcoma and Spindle Cell Sarcoma of Bone. BMC Cancer.

[25] Lagmay J.P., Krailo M.D., Dang H., Kim A., Hawkins D.S., Beaty III O., Janeway K.A. (2016). Outcome of Patients with Recurrent Osteosarcoma Enrolled in Seven Phase II Trials Through Children’s Cancer Group, Pediatric Oncology Group, and Children’s Oncology Group: Learning From the Past to Move Forward. J. Clin. Oncol..

[26] Arndt C.A., Koshkina N.V., Inwards C.Y., Hawkins D.S., Krailo M.D., Villaluna D., Anderson P.M., Goorin A.M., Blakely M.L., Bernstein M. (2010). Inhaled Granulocyte-Macrophage Colony Stimulating Factor for First Pulmonary Recurrence of Osteosarcoma: Effects on Disease-Free Survival and Immunomodulation. A Report From the Children’s Oncology Group. Clin. Cancer Res..

[27] Marina N.M., Smeland S., Bielack S.S., Bernstein M., Jovic G., Krailo M.D., Hook J.M., Arndt C., van den Berg H., Brennan B. (2016). Comparison of MAPIE versus MAP in Patients with a Poor Response to Preoperative Chemotherapy for Newly Diagnosed High-Grade Osteosarcoma (EURAMOS-1): An Open-Label, International, Randomised Controlled Trial. Lancet Oncol..

[28] Smrke A., Anderson P., Gulia A., Gennatas S., Huang P., Jones R. (2021). Future Directions in the Treatment of Osteosarcoma. Cells.

[29] Lettieri C.K., Appel N., Labban N., Lussier D.M., Blattman J.N., Hingorani P. (2016). Progress and Opportunities for Immune Therapeutics in Osteosarcoma. Immunotherapy.

[30] Wang Z., Li B., Ren Y., Ye Z. (2016). T-Cell-Based Immunotherapy for Osteosarcoma: Challenges and Opportunities. Front. Immunol..

[31] DeMaria O., Cornen S., Daëron M., Morel Y., Medzhitov R., Vivier E. (2019). Harnessing Innate Immunity in Cancer Therapy. Nature.

[32] Corrales L., Matson V., Flood B., Spranger S., Gajewski T.F. (2016). Innate Immune Signaling and Regulation in Cancer Immunotherapy. Cell Res..

[33] Koirala P., Roth M.E., Gill J., Piperdi S., Chinai J.M., Geller D.S., Hoang B.H., Park A., Fremed M.A., Zang X. (2016). Immune infiltration and PD-L1 Expression in the Tumor Microenvironment Are Prognostic in Osteosarcoma. Sci. Rep..

[34] Corre I., Verrecchia F., Crenn V., Redini F., Trichet V. (2020). The Osteosarcoma Microenvironment: A Complex but Targetable Ecosystem. Cells.

[35] Yoshida K., Okamoto M., Sasaki J., Kuroda C., Ishida H., Ueda K., Ideta H., Kamanaka T., Sobajima A., Takizawa T. (2020). Anti-PD-1 Antibody Decreases Tumour-Infiltrating Regulatory T cells. BMC Cancer.

[36] Wedekind M.F., Wagner L.M., Cripe T.P. (2018). Immunotherapy for Osteosarcoma: Where Do We Go from Here?. Pediatr. Blood Cancer.

[37] El-Naggar A.M., Clarkson P.W., Negri G.L., Turgu B., Zhang F., Anglesio M.S., Sorensen P.H. (2019). HACE1 is a Potential Tumor Suppressor in Osteosarcoma. Cell Death Dis..

[38] Forrest S.J., Kinnaman M.D., Livingston J.A., Vo K.T., Merriam P., Clinton C., Desmith K., Cavanaugh K., Felicetti B., Smith S. (2021). Phase II Trial of Olaparib in Combination with Ceralasertib in Patients with Recurrent Osteosarcoma. J. Clin. Oncol..

[39] Bousquet M., Noirot C., Accadbled F., de Gauzy J.S., Castex M., Brousset P., Gomez-Brouchet A. (2016). Whole-Exome Sequencing in Osteosarcoma Reveals Important Heterogeneity of Genetic Alterations. Ann. Oncol..

[40] Ho X.D., Phung P., Le V.Q., Nguyen V.H., Reimann E., Prans E., Kõks G., Maasalu K., Le N.T., Trinh L.H. (2017). Whole Transcriptome Analysis Identifies Differentially Regulated Networks between Osteosarcoma and Normal Bone Samples. Exp. Biol. Med..

[41] Joseph C.G., Hwang H., Jiao Y., Wood L.D., Kinde I., Wu J., Mandahl N., Luo J., Hruban R.H., Diaz L. (2013). Exomic Analysis of Myxoid Liposarcomas, Synovial Sarcomas, and Osteosarcomas. Genes. Chromosom. Cancer.

[42] Kiezun A., Perry J., Tonzi P., Van Allen E., Carter S.L., Baca S., Bhatt A., Lawrence M., Walensky L., Wagle N. (2014). Abstract A41: Complementary Genomic Approaches Highlight the PI3K/mTOR Pathway as a Common Vulnerability in Osteosarcoma. Cancer Res..

[43] Behjati S., Tarpey P.S., Haase K., Ye H., Young M.D., Alexandrov L.B., Farndon S.J., Collord G., Wedge D.C., Martincorena I. (2017). Recurrent Mutation of IGF Signalling Genes and Distinct Patterns of Genomic Rearrangement in Osteosarcoma. Nat. Commun..

[44] Matsuoka K., Bakiri L., Wolff L.I., Linder M., Mikels-Vigdal A., Patiño-García A., Lecanda F., Hartmann C., Sibilia M., Wagner E.F. (2020). Wnt Signaling and Loxl2 Promote Aggressive Osteosarcoma. Cell Res..

[45] Feng W., Dean D.C., Hornicek F.J., Spentzos D., Hoffman R.M., Shi H., Duan Z. (2020). Myc is a Prognostic Biomarker and Potential Therapeutic Target in Osteosarcoma. Ther. Adv. Med Oncol..

[46] Kovac M., Blattmann C., Ribi S., Smida J., Mueller N.S., Engert F., Castro-Giner F., Weischenfeldt J., Kovacova M., Krieg A. (2015). Exome Sequencing of Osteosarcoma Reveals Mutation Signatures Reminiscent of Brca Deficiency. Nat. Commun..

[47] Ma X., Liu Y., Liu Y., Alexandrov L.B., Edmonson M.N., Gawad C., Zhou X., Li Y., Rusch M.C., Easton J. (2018). Pan-Cancer Genome and Transcriptome Analyses of 1699 Paediatric Leukaemias and Solid Tumours. Nature.

[48] Chen X., Bahrami A., Pappo A., Easton J., Dalton J., Hedlund E., Ellison D., Shurtleff S., Wu G., Wei L. (2014). Recurrent Somatic Structural Variations Contribute to Tumorigenesis in Pediatric Osteosarcoma. Cell Rep..

[49] Engeland K. (2022). Cell Cycle Regulation: P53-P21-Rb Signaling. Cell Death Differ..

[50] Lu L., Jin W., Wang L.L. (2020). RECQ DNA Helicases and Osteosarcoma. Curr. Adv. Sci. Osteosarcoma.

[51] Mo D., Zhao Y., Balajee A.S. (2017). Human RecQL4 Helicase Plays Multifaceted Roles in the Genomic Stability of Normal and Cancer Cells. Cancer Lett..

[52] Mirabello L., Zhu B., Koster R., Karlins E., Dean M., Yeager M., Gianferante M., Spector L.G., Morton L.M., Karyadi D. (2020). Frequency of Pathogenic Germline Variants in Cancer-Susceptibility Genes in Patients With Osteosarcoma. JAMA Oncol..

[53] Rickel K., Fang F., Tao J. (2017). Molecular Genetics of Osteosarcoma. Bone.

[54] Farmer H., McCabe N., Lord C.J., Tutt A.N.J., Johnson D.A., Richardson T.B., Santarosa M., Dillon K.J., Hickson I., Knights C. (2005). Targeting the DNA Repair Defect in BRCA Mutant Cells as a Therapeutic Strategy. Nature.

[55] Kohlmeyer J.L., Gordon D.J., Tanas M.R., Monga V., Dodd R.D., Quelle D.E. (2020). CDKs in Sarcoma: Mediators of Disease and Emerging Therapeutic Targets. Int. J. Mol. Sci..

[56] Ragland B.D., Bell W.C., Lopez R.R., Siegal G.P. (2002). Cytogenetics and Molecular Biology of Osteosarcoma. Lab. Investig..

[57] Czarnecka A.M., Synoradzki K., Firlej W., Bartnik E., Sobczuk P., Fiedorowicz M., Grieb P., Rutkowski P. (2020). Molecular Biology of Osteosarcoma. Cancers.

[58] Carter J.M., Inwards C.Y., Jin L., Evers B., Wenger D.E., Oliveira A.M., Fritchie K.J. (2014). Activating GNAS Mutations in Parosteal Osteosarcoma. Am. J. Surg. Pathol..

[59] Jackson S.P., Bartek J. (2009). The DNA-Damage Response in Human Biology and Disease. Nature.

[60] Geenen J.J.J., Schellens J.H.M. (2017). Molecular Pathways: Targeting the Protein Kinase Wee1 in Cancer. Clin. Cancer Res..

[61] Kolb E.A., Houghton P.J., Kurmasheva R.T., Mosse Y.P., Maris J.M., Erickson S.W., Guo Y., Teicher B.A., Smith M.A., Gorlick R. (2020). Preclinical Evaluation of the Combination of AZD1775 and Irinotecan against Selected Pediatric Solid Tumors: A Pediatric Preclinical Testing Consortium report. Pediatr. Blood Cancer.

[62] Matheson C.J., Backos D.S., Reigan P. (2016). Targeting WEE1 Kinase in Cancer. Trends Pharmacol. Sci..

[63] Gill J., Gorlick R. (2021). Advancing Therapy for Osteosarcoma. Nat. Rev. Clin. Oncol..

[64] Sayles L.C., Breese M.R., Koehne A.L., Leung S.G., Lee A.G., Liu H.-Y., Spillinger A., Shah A.T., Tanasa B., Straessler K. (2019). Genome-Informed Targeted Therapy for Osteosarcoma. Cancer Discov..

[65] Zhou Y., Shen J.K., Yu Z., Hornicek F.J., Kan Q., Duan Z. (2018). Expression and Therapeutic Implications of Cyclin-Dependent Kinase 4 (CDK4) in Osteosarcoma. Biochim. Biophys. Acta (BBA)—Mol. Basis Dis..

[66] Higuchi T., Sugisawa N., Miyake K., Oshiro H., Yamamoto N., Hayashi K., Kimura H., Miwa S., Igarashi K., Chawla S.P. (2019). Sorafenib and Palbociclib Combination Regresses a Cisplatinum-Resistant Osteosarcoma in a PDOX Mouse Model. Anticancer Res..

[67] Fu W., Sharma S.S., Ma L., Chu B., Bui M.M., Reed D., Pledger W.J. (2013). Apoptosis of Osteosarcoma Cultures by the Combination of the Cyclin-Dependent Kinase Inhibitor SCH727965 and a Heat Shock Protein 90 Inhibitor. Cell Death Dis..

[68] Tavanti E., Sero V., Vella S., Fanelli M., Michelacci F., Landuzzi L., Magagnoli G., Versteeg R., Picci P., Hattinger C. (2013). Preclinical Validation of Aurora Kinases-Targeting Drugs in Osteosarcoma. Br. J. Cancer.

[69] Zhou S.-F., Niu N.-K., Wang Z.-L., Pan S.-T., Ding H.-Q., Au G.H.T., He Z.-X., Zhou Z.-W., Xiao G., Yang Y.-X. (2015). Pro-Apoptotic and Pro-Autophagic Effects of the Aurora Kinase A Inhibitor Alisertib (MLN8237) on Human Osteosarcoma U-2 OS and MG-63 Cells through the Activation of Mitochondria-Mediated Pathway and Inhibition of p38 MAPK/PI3K/Akt/mTOR Signaling Pathway. Drug Des. Dev. Ther..

[70] Zhao Z., Jin G., Yao K., Liu K., Liu F., Chen H., Wang K., Gorja D.R., Reddy K., Bode A.M. (2019). Aurora B Kinase as a Novel Molecular Target for Inhibition the Growth of Osteosarcoma. Mol. Carcinog..

[71] Wu C.-C., Livingston J.A. (2020). Genomics and the Immune Landscape of Osteosarcoma. Curr. Adv. Sci. Osteosarcoma.

[72] Assi T., Watson S., Samra B., Rassy E., Le Cesne A., Italiano A., Mir O. (2021). Targeting the VEGF Pathway in Osteosarcoma. Cells.

[73] Yang J., Yang D., Sun Y., Sun B., Wang G., Trent J.C., Araujo D.M., Chen K., Zhang W. (2011). Genetic Amplification of the Vascular Endothelial Growth Factor (VEGF) Pathway Genes, Including *VEGFA*, in Human Osteosarcoma. Cancer.

[74] Yu X.W., Wu T.Y., Yi X., Ren W.P., Zhou Z.B., Sun Y.Q., Zhang C.Q. (2014). Prognostic Significance of VEGF Expression in Osteosarcoma: A Meta-Analysis. Tumour Biol..

[75] Italiano A., Mir O., Mathoulin-Pelissier S., Penel N., Piperno-Neumann S., Bompas E., Chevreau C., Duffaud F., Entz-Werlé N., Saada E. (2020). Cabozantinib in Patients with Advanced Ewing Sarcoma or Osteosarcoma (CABONE): A Multicentre, Single-Arm, Phase 2 Trial. Lancet Oncol..

[76] Gaspar N., Casanova M., Sirvent F.J.B., Venkatramani R., Morland B., Gambart M., Thebaud E., Strauss S.J., Locatelli F., Melcon S.G. (2018). Single-Agent Expansion Cohort of Lenvatinib (Len) and Combination Dose-Finding Cohort of LEN + Etoposide (ETP) + Ifosfamide (IFM) in Patients (pts) Aged 2 to ≤25 Years with Relapsed/Refractory Osteosarcoma (OS). J. Clin. Oncol..

[77] Gaspar N., Campbell-Hewson Q., Melcon S.G., Locatelli F., Venkatramani R., Hecker-Nolting S., Gambart M., Bautista F., Thebaud E., Aerts I. (2021). Phase I/II Study of Single-Agent Lenvatinib in Children and Adolescents with Refractory or Relapsed Solid Malignancies and Young Adults with Osteosarcoma (ITCC-050)☆. ESMO Open.

[78] Duffaud F., Mir O., Boudou-Rouquette P., Piperno-Neumann S., Penel N., Bompas E., Delcambre C., Kalbacher E., Italiano A., Collard O. (2018). Efficacy and Safety of Regorafenib in Adult Patients with Metastatic Osteosarcoma: A Non-Comparative, Randomised, Double-Blind, Placebo-Controlled, Phase 2 Study. Lancet Oncol..

[79] Davis L.E., Bolejack V., Ryan C.W., Ganjoo K.N., Loggers E.T., Chawla S., Agulnik M., Livingston M.B., Reed D., Keedy V. (2019). Randomized Double-Blind Phase II Study of Regorafenib in Patients With Metastatic Osteosarcoma. J. Clin. Oncol..

[80] Li X., Pontén A., Aase K., Karlsson L., Abramsson A., Uutela M., Bäckström G., Hellström M., Boström H., Li H. (2000). PDGF-C Is a New Protease-Activated Ligand for the PDGF α-Receptor. Nat. Cell Biol..

[81] LaRochelle W.J., Jeffers M., McDonald W.F., Chillakuru R.A., Giese N.A., Lokker N.A., Sullivan C., Boldog F.L., Yang M., Vernet C. (2001). PDGF-D, a New Protease-Activated Growth Factor. Nat. Cell Biol..

[82] Bartoschek M., Pietras K. (2018). PDGF Family Function and Prognostic Value in Tumor Biology. Biochem. Biophys. Res. Commun..

[83] Heldin C.-H., Lennartsson J., Westermark B. (2018). Involvement of Platelet-Derived Growth Factor Ligands and Receptors in Tumorigenesis. J. Intern. Med..

[84] Kubo T., Bs S.P., Rosenblum J., Antonescu C.R., Chen W., Kim H.-S., Huvos A.G., Bs R.S., Meyers P.A., Healey J.H. (2008). Platelet-Derived Growth Factor Receptor as a Prognostic Marker and a Therapeutic Target for Imatinib Mesylate Therapy in Osteosarcoma. Cancer.

[85] Fernandes I., Melo-Alvim C., Lopes-Brás R., Esperança-Martins M., Costa L. (2021). Osteosarcoma Pathogenesis Leads the Way to New Target Treatments. Int. J. Mol. Sci..

[86] Yamaguchi S.I., Ueki A., Sugihara E., Onishi N., Yaguchi T., Kawakami Y., Horiuchi K., Morioka H., Matsumoto M., Nakamura M. (2015). Synergistic Antiproliferative Effect of Imatinib and Adriamycin in Platelet-Derived Growth Factor Receptor-Expressing Osteosarcoma Cells. Cancer Sci..

[87] Papadopoulos N., Lennartsson J. (2018). The PDGF/PDGFR Pathway as a Drug Target. Mol. Asp. Med..

[88] Shen G., Zheng F., Ren D., Du F., Dong Q., Wang Z., Zhao F., Ahmad R., Zhao J. (2018). Anlotinib: A Novel Multi-Targeting Tyrosine Kinase Inhibitor in Clinical Development. J. Hematol. Oncol..

[89] Kumar R.M.R., Arlt M.J., Kuzmanov A., Born W., Fuchs B. (2015). Sunitinib Malate (SU-11248) Reduces Tumour Burden and Lung Metastasis in an Intratibial Human Xenograft Osteosarcoma Mouse Model. Am. J. Cancer Res..

[90] McCarthy T.L., Centrella M. (2001). Local IGF-I Expression and Bone Formation. Growth Horm. IGF Res..

[91] Majidinia M., Sadeghpour A., Yousefi B. (2018). The Roles of Signaling Pathways in Bone Repair and Regeneration. J. Cell. Physiol..

[92] Mancarella C., Morrione A., Scotlandi K. (2021). Unraveling the IGF System Interactome in Sarcomas Exploits Novel Therapeutic Options. Cells.

[93] Tzanakakis G., Giatagana E.-M., Berdiaki A., Spyridaki I., Hida K., Neagu M., Tsatsakis A., Nikitovic D. (2021). The Role of IGF/IGF-IR-Signaling and Extracellular Matrix Effectors in Bone Sarcoma Pathogenesis. Cancers.

[94] Chmielowski B. (2014). Insulin-Like Growth Factor 1 Receptor Inhibitors: Where Do We Come from? What Are We? Where Are We Going?. Cancer.

[95] Cohen D.H., Leroith D. (2012). Obesity, Type 2 Diabetes and Cancer: The Insulin and IGF Connection. Endocr.-Relat. Cancer.

[96] Cao J., Yee D. (2021). Disrupting Insulin and IGF Receptor Function in Cancer. Int. J. Mol. Sci..

[97] Wang Y.-H., Han X.-D., Qiu Y., Xiong J., Yu Y., Wang B., Zhu Z.-Z., Qian B.-P., Chen Y.-X., Wang S.-F. (2011). Increased Expression of Insulin-Like Growth Factor-1 Receptor is Correlated with Tumor Metastasis and Prognosis in Patients with Osteosarcoma. J. Surg. Oncol..

[98] Morrow J.J., Bayles I., Funnell A.P.W., Miller T.E., Saiakhova A., Lizardo M.M., Bartels C.F., Kapteijn M.Y., Hung S., Mendoza A. (2018). Positively Selected Enhancer Elements Endow Osteosarcoma Cells with Metastatic Competence. Nat. Med..

[99] Goudarzi A., Gokgoz N., Gill M., Pinnaduwage D., Merico D., Wunder J.S., Andrulis I.L. (2013). Protein Kinase C Epsilon and Genetic Networks in Osteosarcoma Metastasis. Cancers.

[100] Mansky P.J., Liewehr D.J., Steinberg S.M., Chrousos G.P., Avila N.A., Long L., Bernstein D., Mackall C.L., Hawkins D.S., Helman L.J. (2002). Treatment of Metastatic Osteosarcoma With the Somatostatin Analog OncoLar: Significant Reduction of Insulin-Like Growth Factor-1 Serum Levels. J. Pediatr. Hematol..

[101] Beck O., Paret C., Russo A., Burhenne J., Fresnais M., Steimel K., Seidmann L., Wagner D.-C., Vewinger N., Lehmann N. (2020). Safety and Activity of the Combination of Ceritinib and Dasatinib in Osteosarcoma. Cancers.

[102] Pappo A.S., Vassal G., Crowley J.J., Bolejack V., Hogendoorn P.C., Chugh R., Ladanyi M., Grippo J.F., Dall G., Staddon A.P. (2014). A Phase 2 Trial of R1507, a Monoclonal Antibody to the Insulin-Like Growth Factor-1 Receptor (IGF-1R), in Patients with Recurrent or Refractory Rhabdomyosarcoma, Osteosarcoma, Synovial Sarcoma, and Other Soft Tissue Sarcomas: Results of a Sarcoma Alliance for Research Through Collaboration study. Cancer.

[103] Hattinger C.M., Pasello M., Ferrari S., Picci P., Serra M. (2010). Emerging Drugs for High-Grade Osteosarcoma. Expert Opin. Emerg. Drugs.

[104] Chawla S.P., Staddon A.P., Baker L.H., Schuetze S.M., Tolcher A.W., D’Amato G.Z., Blay J.-Y., Mita M.M., Sankhala K.K., Berk L. (2012). Phase II Study of the Mammalian Target of Rapamycin Inhibitor Ridaforolimus in Patients With Advanced Bone and Soft Tissue Sarcomas. J. Clin. Oncol..

[105] Gazitt Y., Kolaparthi V., Moncada K., Thomas C., Freeman J. (1992). Targeted Therapy of Human Osteosarcoma with 17AAG or Rapamycin: Characterization of Induced Apoptosis and Inhibition of mTOR and Akt/MAPK/Wnt Pathways. Int. J. Oncol..

[106] Ding L., Congwei L., Bei Q., Tao Y., Ruiguo W., Heze Y., Bo D., Zhihong L. (2016). Mtor: An Attractive Therapeutic Target for Osteosarcoma?. Oncotarget.

[107] Gobin B., Battaglia S., Lanel R., Chesneau J., Amiaud J., Rédini F., Ory B., Heymann D. (2014). NVP-BEZ235, a Dual PI3K/mTOR Inhibitor, Inhibits Osteosarcoma Cell Proliferation and Tumor Development in Vivo with an Improved Survival Rate. Cancer Lett..

[108] Zhu Y.-R., Min H., Fang J.-F., Zhou F., Deng X.-W., Zhang Y.-Q. (2015). Activity of the Novel Dual Phosphatidylinositol 3-Kinase/Mammalian Target of Rapamycin Inhibitor NVP-BEZ235 against Osteosarcoma. Cancer Biol. Ther..

[109] Bavelloni A., Focaccia E., Piazzi M., Orsini A., Ramazzotti G., Cocco L., Blalock W., Faenza I. (2018). Therapeutic Potential of Nvp-bkm120 in Human Osteosarcomas Cells. J. Cell. Physiol..

[110] Yu W.-X., Lu C., Wang B., Ren X.-Y., Xu K. (2020). Effects of Rapamycin on Osteosarcoma Cell Proliferation and Apoptosis by Inducing Autophagy. Eur. Rev. Med. Pharmacol. Sci..

[111] Oshiro H., Tome Y., Miyake K., Higuchi T., Sugisawa N., Kanaya F., Nishida K., Hoffman R.M. (2021). An mTOR and VEGFR Inhibitor Combination Arrests a Doxorubicin Resistant Lung Metastatic Osteosarcoma in a PDOX Mouse Model. Sci. Rep..

[112] Zheng C., Tang F., Min L., Hornicek F., Duan Z., Tu C. (2020). PTEN in Osteosarcoma: Recent Advances and the Therapeutic Potential. Biochim. Biophys. Acta.

[113] Li H., Shen X., Ma M., Liu W., Yang W., Wang P., Cai Z., Mi R., Lu Y., Zhuang J. (2021). ZIP10 Drives Osteosarcoma Proliferation and Chemoresistance through ITGA10-Mediated Activation of the PI3K/AKT Pathway. J. Exp. Clin. Cancer Res..

[114] Dang C.V., O’Donnell K.A., Zeller K.I., Nguyen T., Osthus R.C., Li F. (2006). The C-Myc Target Gene Network. Semin. Cancer Biol..

[115] Dhanasekaran R., Deutzmann A., Mahauad-Fernandez W.D., Hansen A.S., Gouw A.M., Felsher D.W. (2021). The MYC Oncogene—The Grand Orchestrator of Cancer Growth and Immune Evasion. Nat. Rev. Clin. Oncol..

[116] Dong Y., Tu R., Liu H., Qing G. (2020). Regulation of Cancer Cell Metabolism: Oncogenic MYC in the Driver’s Seat. Signal Transduct. Target. Ther..

[117] Dang C.V. (2012). MYC on the Path to Cancer. Cell.

[118] Baluapuri A., Wolf E., Eilers M. (2020). Target Gene-Independent Functions of MYC Oncoproteins. Nat. Rev. Mol. Cell Biol..

[119] Han G., Wang Y., Bi W. (2012). C-Myc Overexpression Promotes Osteosarcoma Cell Invasion via Activation of MEK-ERK Pathway. Oncol. Res. Featur. Preclin. Clin. Cancer Ther..

[120] Chen D., Zhao Z., Huang Z., Chen D.C., Zhu X.X., Wang Y.Z., Yan Y.W., Tang S., Madhavan S., Ni W. (2018). Super enhancer inhibitors suppress MYC driven transcriptional amplification and tumor progression in osteosarcoma. Bone Res..

[121] Lee D.H., Qi J., Bradner J.E., Said J.W., Doan N.B., Forscher C., Yang H., Koeffler H.P. (2014). Synergistic Effect of JQ1 and Rapamycin for Treatment of Human Osteosarcoma. Int. J. Cancer.

[122] Roth M., Barris D.M., Piperdi S., Kuo V., Everts S., Geller D., Houghton P., Kolb E.A., Hawthorne T., Gill J. (2015). Targeting Glycoprotein NMB with Antibody-Drug Conjugate, Glembatumumab Vedotin, for the Treatment of Osteosarcoma. Pediatr. Blood Cancer.

[123] Wang L., Zhang Q., Chen W., Shan B., Ding Y., Zhang G., Cao N., Liu L., Zhang Y. (2013). B7-H3 is Overexpressed in Patients Suffering Osteosarcoma and Associated with Tumor Aggressiveness and Metastasis. PLoS ONE.

[124] Cui J., Dean D., Wei R., Hornicek F.J., Ulmert D., Duan Z. (2020). Expression and Clinical Implications of Leucine-Rich Repeat Containing 15 (LRRC15) in Osteosarcoma. J. Orthop. Res..

[125] Kopp L.M., Malempati S., Krailo M., Gao Y., Buxton A., Weigel B.J., Hawthorne T., Crowley E., Moscow J.A., Hawthorne T. (2019). Phase II Trial of the Glycoprotein Non-Metastatic B-Targeted Antibody-Drug Conjugate, Glembatumumab Vedotin (CDX-011), in Recurrent Osteosarcoma AOST1521: A Report from the Children’s Oncology Group. Eur. J. Cancer.

[126] Hingorani P., Roth M.E., Wang Y., Zhang W., Gill J.B., Harrison D.J., Teicher B., Erickson S., Gatto G., Smith M.A. (2021). ABBV-085, Antibody–Drug Conjugate Targeting LRRC15, Is Effective in Osteosarcoma: A Report by the Pediatric Preclinical Testing Consortium. Mol. Cancer Ther..

[127] Park J.A., Cheung N.-K.V. (2020). GD2 or HER2 Targeting T Cell Engaging Bispecific Antibodies to Treat Osteosarcoma. J. Hematol. Oncol..

[128] Agostinis P., Berg K., Cengel K.A., Foster T.H., Girotti A.W., Gollnick S.O., Hahn S.M., Hamblin M.R., Juzeniene A., Kessel D. (2011). Photodynamic Therapy of Cancer: An Update. CA Cancer J. Clin..

[129] Nakamura T., Kusuzaki K., Matsubara T., Matsumine A., Murata H., Uchida A. (2008). A New Limb Salvage Surgery in Cases of High-Grade Soft Tissue Sarcoma Using Photodynamic Surgery, Followed by Photo and Radiodynamic Therapy with Acridine Orange. J. Surg. Oncol..

[130] Yu W., Zhu J., Wang Y., Wang J., Fang W., Xia K., Shao J., Wu M., Liu B., Liang C. (2017). A Review and Outlook in the Treatment of Osteosarcoma and Other Deep Tumors with Photodynamic Therapy: From Basic to Deep. Oncotarget.

[131] Tan G., Xu J., Yu Q., Yang Z., Zhang H. (2022). The Safety and Efficiency of Photodynamic Therapy for the Treatment of Osteosarcoma: A Systematic Review of in Vitro Experiment and Animal Model Reports. Photodiagnosis Photodyn. Ther..

[132] Tang Z., Zhao L., Yang Z., Liu Z., Gu J., Bai B., Liu J., Xu J., Yang H. (2018). Mechanisms of Oxidative Stress, Apoptosis, and Autophagy Involved in Graphene Oxide Nanomaterial Anti-Osteosarcoma Effect. Int. J. Nanomed..

[133] Barani M., Mukhtar M., Rahdar A., Sargazi S., Pandey S., Kang M. (2021). Recent Advances in Nanotechnology-Based Diagnosis and Treatments of Human Osteosarcoma. Biosensors.

[134] Yao M., Ma L., Li L., Zhang J., Lim R.X., Chen W., Zhang Y. (2016). A New Modality for Cancer Treatment—Nanoparticle Mediated Microwave Induced Photodynamic Therapy. J. Biomed. Nanotechnol..

[135] Yu W., Ye M., Zhu J., Wang Y., Liang C., Tang J., Tao H., Shen Y. (2018). Zinc Phthalocyanine Encapsulated in Polymer Micelles as a Potent Photosensitizer for the Photodynamic Therapy of Osteosarcoma. Nanomed. Nanotechnol. Biol. Med..

[136] Gao Z., Zhang L., Hu J., Sun Y. (2013). Mesenchymal Stem Cells: A Potential Targeted-Delivery Vehicle for Anti-Cancer Drug Loaded Nanoparticles. Nanomed. Nanotechnol. Biol. Med..

[137] Layek B., Sadhukha T., Panyam J., Prabha S. (2018). Nano-Engineered Mesenchymal Stem Cells Increase Therapeutic Efficacy of Anticancer Drug Through True Active Tumor Targeting. Mol. Cancer Ther..

[138] Lenna S., Bellotti C., Duchi S., Martella E., Columbaro M., Dozza B., Ballestri M., Guerrini A., Sotgiu G., Frisoni T. (2020). Mesenchymal Stromal Cells Mediated Delivery of Photoactive Nanoparticles Inhibits Osteosarcoma Growth in Vitro and in a Murine in Vivo Ectopic Model. J. Exp. Clin. Cancer Res..

[139] Martin J.W., Squire J., Zielenska M. (2012). The Genetics of Osteosarcoma. Sarcoma.

[140] Li F.P., Fraumeni J.F. (1969). Soft-Tissue Sarcomas, Breast Cancer, and Other Neoplasms. A Familial Syndrome?. Ann. Intern. Med..

[141] Moukengue B., Lallier M., Marchandet L., Baud’Huin M., Verrecchia F., Ory B., Lamoureux F. (2022). Origin and Therapies of Osteosarcoma. Cancers.

[142] Zhang L., Li Y., Hu C., Chen Y., Chen Z., Chen Z.S., Zhang J.Y., Fang S. (2022). CDK6-PI3K Signaling Axis is an Efficient Target for Attenuating ABCB1/P-Gp Mediated Multi-Drug Resistance (MDR) in Cancer Cells. Mol. Cancer.

[143] Chaudhary S., Pothuraju R., Rachagani S., Siddiqui J.A., Atri P., Mallya K., Nasser M.W., Sayed Z., Lyden E.R., Smith L. (2021). Dual Blockade of EGFR and CDK4/6 Delays Head and Neck Squamous Cell Carcinoma Progression by Inducing Metabolic Rewiring. Cancer Lett..

[144] Jiang Z.-Y., Liu J.-B., Wang X.-F., Ma Y.-S., Fu D. (2022). Current Status and Prospects of Clinical Treatment of Osteosarcoma. Technol. Cancer Res. Treat..

[145] Xie D., Wang Z., Li J., Guo D.-A., Lu A., Liang C. (2022). Targeted Delivery of Chemotherapeutic Agents for Osteosarcoma Treatment. Front. Oncol..

